# Unqualified Medical Practice

**Published:** 1917-10-06

**Authors:** 


### UNQUALIFIED MEDICAL PRACTICE.

### Should it be Suppressed by Law?

The Annual Report of the Medical Defence Union is always a document of interest. The issue for 1916 presents a picture of what may be called the difficulties and trials of medical practice as these are provided by the human relations which such practice involves. The scientific and practical problems of diagnosis and treatment are anxious enough in all conscience, but they have also their stimulating aspect, and are, in any event, an essential part of the enterprise. For their solution the practitioner has to depend on his technical equipment and educated judgment, and the discipline has its compensations. There are, however, vexations and annoyances and worries which arrive from personal misunderstandings and misconceptions, sometimes on the part of patients, sometimes on the part of colleagues, and to meet these successfully there is need for another order of training than that which is provided in the medical curriculum. The practitioner is necessarily dealing with a public which cannot fully appreciate the motives which direct the medical art, and which is apt to expect more than this art professes to accomplish.

### The Risk of Slander.

Even more serious is the consideration that not only misrepresentation, but actual slander may be the practitioner's fate. This is the more grave as the discovery of its authors is not always an easy task, and confidence may be sapped by vague hints and suggestions which stop short of a definite proposition. In some degree every citizen comes under this risk, but it weighs particularly on the practitioner of medicine, for the basis of his work is public confidence, and public confidence depends on reputation. Hence misrepresentation and slander mean for the practitioner not only a question of self-respect, but also a question of livelihood. One may fondly hope to avoid all these possibilities, but it is an old lesson, "Be thou as chaste as ice, as pure as snow, thou shalt not escape calumny." In short, so long as among sick people and their friends there are stupid, wrong-headed, and mischief-making folk, medical practice will involve personal annoyances and dangers for the practitioner. In spite of himself, he will at times be driven to take measures for the protection of his character and the assertion of his legal rights, and in taking such measures he will need to walk warily and to choose for his counsellors prudence, discretion, good judgment, and worldly wisdom. Who is sufficient for these things? Certainly not the practitioner immersed in the anxieties and cares of his suffering neighbours, and irritated by comments and innuendoes which he knows to be unjust and absurd, if not deliberately wicked. He is struck in what is most dear to him—namely, his personal and professional reputation, and the blow is sometimes delivered by an unseen hand. It is hardly to be wondered at that in such circumstances his anger and indignation hurry him into methods which are unwise, and which are subsequently found to entangle and embitter a situation that in its original form might have been easily adjusted.

### Value of Union.

Now what the Medical Defence Union takes for its sphere is the prevention of these unhappy and troublesome developments. Each member of the Union has at his elbow, as one may say, a judicious and experienced counsellor who has lived through all the varieties of storm which are apt to break over the head of the practitioner of medicine. He may learn by a personal interview, or even by correspondence, what exactly he ought to do and what he ought to abstain from doing. Instead of agitating himself by internal and domestic debates on what is the best way of speaking to his enemy in the gate, he is able to commit all his case to a powerful and experienced and well-equipped organisation, and to know that all possible measures will be taken for the protection of his interests. The contrast between such a poistion and that of an individual—wronged, angry, and irritated—seeking redress according to his own judgment (or the lack of it) is a very vivid one, and it ought to command the appreciation of every member of the profession. Nor is the relief merely mental and emotional. On the contrary, it penetrates the entire position, and saves the pocket as well as spares the cerebral tissues. In other words, whatever expense is necessary to secure satisfaction and protection is undertaken by the Union, and the member has no terrors of a possible bill of costs.

Again, the isolated and unprotected practitioner is a ready prey to the designing person who proposes an action for malpraxis as an answer to a demand for the payment of fees, and he occupies much the same position in relation to the speculative solicitor, who, low be it spoken, is to be found in spite of the refining atmosphere of legal procedure. But when the practitioner appears as part and parcel of a powerful organisation, the enemy is more likely to display discretion rather than valour. Now it is recognised that the true position will be fully explored, that knowledge, experience, and good judgment will have to be met, and that funds ready for any legal enterprise are readily available. Many a loud-voiced and noisy suitor, when introduced to this formidable apparatus, has recognised that the game is up, and has been glad to listen to reason and to common sense or to still more persuasive arguments.

### The Path of Prudence.

Such, in brief outline, are the advantages which are offered by membership of the Medical Defence Union. They are set forth year after year; and yet there are those who heed them not. The dangers exist, as each man must allow, and protection is desirable. But some there be who act as though these truths do not apply to themselves. This comfortable conviction is now and again rudely disturbed, and the man who has for years dwelt peacefully under his own vine and fig-tree awakens to find himself involved in mischiefs and miseries against which he is ill-prepared to struggle. All men of prudence will avoid this possibility, and they can do so by the small annual charge which is one of the conditions of membership of the Medical Defence Union. The sum is small, the relief is great, the protection sure. Wisdom and economy speak here with a single voice, and those who will not hear risk trouble and loss. Even if the risk is never realised, there is abundant compensation and much mental comfort in the thought that the shield is ready should its protective security ever be required.

### Assumption of Medical Titles.

While the main work of the Union is the protection of the interests of the individual practitioner whenever these are invaded or challenged, it aspires also to a wider ambition—namely, to the suppression of persons who assume medical titles to which they have no right. As such persons are impostors, and as the law of the land provides a penalty for their deceit, all law-abiding citizens may applaud the Union's efforts in this direction. What Parliament has desired is that the public shall be enabled to distinguish between advisers who have been properly trained and other persons who offer advice detached from such training. Hence the latter are forbidden to employ any style or title which suggests that they possess a diploma as the outcome of a legal curriculum and examination. Any person who makes a profession of this order without warrant is manifestly a sham and a fraud, and the Medical Defence Union does well when it secures his punishment.

### The Limits of Legislation.

But unqualified practice which manifestly and openly professes itself to be what it really is, suffers no challenge from the State. It may be presumption on the part of the adviser and folly on the part of the patient. But it is not illegal. The Medical Defence Union, to judge from its present Report, desires to make it illegal, and it cherishes the hope that " after the war " success in this direction may crown its efforts. We venture to think that this fond anticipation will not be realised. Of the mischiefs and evils of unqualified practice we are fully convinced, and the sham and humbug and vulgarity which distinguish a large part of it merit unmitigated contempt. But that within any future which we can see the Legislature will be led to decree that no one shall be allowed to give medical advice except duly qualified medical practitioners, seems to us an anticipation destitute of all substantial basis. To make clear the true position between advisers who have been trained and those who have not been trained is indeed the function of the Legislature, for this is to protect the public from deceit while leaving open the opportunity for individual choice. But to say that no such choice shall be allowed is quite another proposition, and a proposition, too, which is a direct invasion of personal freedom. Is there any body of public opinion which would urge such a proposal? Surely the answer to this question is decidedly in the negative. Quite naturally and reasonably doctors condemn unqualified practice because they know the risks which it involves, and they have a healthy scorn for the monstrous pretensions and ignorant boasts which characterise it. They are familiar, too, with the mischances and disasters which fall upon a certain proportion of its victims, and with the greedy and unscrupulous proceedings of many of its exponents. Necessarily they must agree that the wise and prudent course is to accept advice only from those who have a sure foundation of technical knowledge and who have been trained in the application of this knowledge. This is what wisdom recommends.

### Need for Freedom of Choice.

But to impose such a policy by legal compulsion needs some sanction other than that provided by prudence and common sense, and the enterprise at once raises the question whether it is better that persons should be driven into wisdom by compulsion, or whether, on the other hand, they should be free to exercise their own judgment. If the first of these alternatives is selected there are few limits to the directing and shepherding action of Government, and not only in medicine but in many other directions prudent courses may be laid down for us by the Legislature. But if freedom of individual choice is the more worthy national ideal we must take it with its disadvantages, and one of these is that a free choice will sometimes be a foolish one. This, on the whole, is the decision which suits best the genius of the British nation, and, *pace* the Medical Defence Union, we do not see any sign that it will be modified in respect to medical practice. Nor do we advocate any such modification. Protect the public most certainly from persons who pretend to technical qualifications which as a matter of fact they do not possess, but do not seek to drive by force of law all patients, reluctant or otherwise, into qualified consulting-rooms. Personally we do not think that the majority of the medical profession desires any such legislative enterprise, and we feel quite sure that neither the Legislature nor the public would suffer it. The Council of the Medical Defence Union obviously has quite another opinion, and we listen to this with great respect. But we cannot accept it, and we fancy the failures which have hitherto marked attempts to put it into practice rest upon circumstances which no professional enthusiasm will be able to overcome. Truth to tell, too, we have no little regard for the independence which resents compulsion even when this is dignified by the association of expert knowledge with admirable intentions. With the general work of the Union we are fully in sympathy, but we cannot go with the ambition to invoke the police and the magistrate as educational agents in the choice of a medical adviser.

